# Applying a deviance framework to understand modern contraceptive use in sub-Saharan Africa

**DOI:** 10.1371/journal.pone.0216381

**Published:** 2019-05-08

**Authors:** Tamar Goldenberg, Rob Stephenson

**Affiliations:** 1 Department of Health Behavior and Health Education, School of Public Health, University of Michigan, Ann Arbor, Michigan, United States of America; 2 Center for Sexuality and Health Disparities, University of Michigan, Ann Arbor, Michigan, United States of America; 3 Department of Systems, Populations and Leadership, School of Nursing, University of Michigan, Ann Arbor, Michigan, United States of America; Tulane University School of Public Health and Tropical Medicine, UNITED STATES

## Abstract

Increasing modern contraceptive use is important for improving maternal and child health and achieving economic growth and development goals. However, pervasive high unmet need for modern contraceptives in sub-Saharan Africa warrants new understandings of the drivers of modern contraceptive use. A deviance approach (i.e., examining how women’s experiences/characteristics differ from other women in their community) provides an innovative framework for capturing heterogeneity among women in a community. This framework can inform public health programming by both exploring how women avoid adverse health outcomes and understanding the needs of harder-to-reach populations who may experience health risks, despite living in communities where others do not experience vulnerability. Using data from Demographic and Health Surveys from 29 sub-Saharan African countries, we examine how a woman’s deviation from community norms around socioeconomic characteristics and gender and fertility norms and behaviors is associated with modern contraceptive use. Random-effects logistic regression models were fitted for each country to examine relationships between modern contraceptive use and deviance. Some deviance factors were associated with modern contraceptive use in only a few countries, while others were significant across many countries. Cross-country consistency in the direction of the relationship between deviance and modern contraceptive use varied by the specific deviance factor, with some relationships being consistent across countries, and other relationships being more varied. For example, having more education than the community norm was associated with increased modern contraceptive use across countries; however, marrying older than other women in the community was associated with an increase in modern contraceptive use in some countries and a decrease in others. More work is needed to understand the role of deviance on modern contraceptive use; however, this study suggests that using context-specific deviance approaches may be important for further elucidating experiences of modern contraceptive use.

## Introduction

Increasing the uptake of modern contraceptive use is an important strategy for improving maternal and child health [1, 2] and is a pathway to achieving developmental goals [3, 4]. In many resource-poor settings, unmet need for modern contraceptives (i.e., the percentage of women who are fecund, sexually-active, and do not want to become pregnant but who are not using modern contraceptives [5]) remains high [6]. In sub-Saharan Africa, rates of modern contraceptive use have increased; however, the overall modern contraceptive prevalence is still low (at 33% in 2015, compared to 64% worldwide), with a high unmet need (24% in 2015, compared with 12% worldwide) [7]. The continuing high unmet need for modern contraceptives in sub-Saharan Africa warrants the need for new understandings of what facilitates or hinders modern contraceptive use in the region.

In recent years, there has been an increased focus on how place shapes modern contraceptive use [8–14]. This research has found that community norms, especially those around fertility and gender, play an important role in facilitating modern contraceptive use. For example, Elfstrom and Stephenson (2012) found that community-level economic prosperity, gender norms, fertility norms, and community health knowledge were associated with modern contraceptive use in 21 African countries and that the effects of these community norms varied by country [11]. Perceptions of community norms may also be important indicators of modern contraceptive use. Dynes et al. (2012) found that in Ethiopia and Kenya, perceptions of a community’s approval of family planning were an important factor associated with contraceptive use [9]. In addition, this study found an association between modern contraceptive use and an individual’s deviation from what they perceived as ideal in the community (e.g., the likelihood of contraceptive use decreased if the perception of a community’s ideal for number of sons was greater than an individual’s actual number of sons). It may not be simply the characteristics of the community environment that shapes an individual’s modern contraceptive use, but also how an individual deviates from their community’s norms and expectations, and how an individual perceives that deviation.

Positive deviance is a useful framework that can be used to capture heterogeneity among women in a community, using a strengths-based lens to understand individuals who do not face the same risk factors for adverse health outcomes as others who are living in their community [15, 16]. A strengths-based lens considers how individuals employ coping mechanisms and avoid adverse health outcomes, even when exposed to potential health risks within their environment. Understanding these experiences of heterogeneity among women in communities builds on Bronfenbrenner’s socio-ecological model [17], which identifies an iterative and reciprocal relationship between an individual and their environment. A deviance framework recognizes that an individual’s experiences and the community context alone may not shape modern contraceptive use, but rather it is the interaction between an individual and their environment. When women deviate from community norms or even from important individuals within their communities, this may influence their health behaviors [18, 19]. An increased understanding of the relationship between positive deviance and modern contraceptive use can inform public health programming and public health interventions by providing insight on how to improve modern contraceptive uptake among other women in the community [20, 21]. This allows us to focus on the resilience that already exists within communities to understand how women are accessing modern contraceptives. At the same time, understanding the relationship between negative deviance and modern contraceptive use can also inform public health programming. Negative deviance refers to individuals who may be exposed to more health risks, despite living in an environment in which other individuals are not necessarily part of a vulnerable group. Understanding negative deviance can inform public health programming by highlighting the needs of harder-to-reach women who may be part of a vulnerable group despite living in communities where most women are not.

At both an individual and community level, many factors have been found to be associated with modern contraceptive use (e.g., socioeconomic characteristics, gender and fertility norms and behaviors) [10, 11, 22–24]; however, the relationships between an individual’s experience with these factors (e.g., age at marriage) and their community’s norms around these factors (e.g., the average age at marriage in a community) is unknown. While it may be expected that positive deviance would be associated with improved health and negative deviance associated with worse health, it is possible that the act of being deviant in either direction may contribute health in different ways; deviance may be perceived as a negative act that isolates a woman from her community, but it may also be an act that helps to increase autonomy and independence. To better understand how deviance from one’s community influences modern contraceptive use, this paper examines the relationships between deviance from community norms and women’s use of modern contraceptives in 29 sub-Saharan African countries. This paper is aligned with the framework of positive and negative deviance; however, in order to not arbitrarily place judgment on what is considered to be a “positive” or “negative” characteristic or behavior, we examine women’s experiences as being more or less than their community norms (e.g., having higher or lower parity).

## Methods

The data used in this analysis are from the women’s questionnaire from the Demographic and Health Surveys (DHS) from 29 countries in sub-Saharan Africa where DHS data were collected in or after 2010. All procedures for DHS surveys have been reviewed and approved by the ICF Institutional Review Board (IRB). All participants provide informed consent. Data can be made available to individuals who apply for use of data to the DHS. For this study, de-identified anonymous data were received after submitting a request.

The DHS uses clustered sampling to collect a nationally-representative sample of ever-married women of reproductive age (ages 15–49). The DHS uses a two-stage sampling design, first creating Primary Sampling Units (PSUs) based on the most recent census data and then interviewing 20–30 households from each PSU.

### Measures

Modern contraceptive use was measured using a binary variable determining whether or not the respondent self-reports currently using a modern method of contraceptives (oral contraceptive pill, IUD, injections, condom, male or female sterilization, implant, or diaphragm/foam/jelly).

Deviance variables were created based on the difference between a woman’s individual response on a given variable and the aggregate community variable. Since community-level variables are not included in the DHS, we created aggregate variables to describe community effects, with each community being defined as one PSU. This is similar to what other studies have done when using DHS data to examine community effects on reproductive health outcomes [11, 25]. Aggregate variables were created by taking the mean (for continuous variables) or proportion (for binary variables) for each variable of all of the responses in a given PSU. To calculate an individual’s deviance from her community, two separate strategies were used for continuous and binary variables. For continuous variables, we first calculated the difference between each woman’s individual characteristic and her community’s mean for that variable. Then, the difference was standardized into z-scores. Women whose individual z-scores were one standard deviation above or below her community’s mean were considered to be deviant. For binary variables, respondents were defined as deviant (having more of a particular characteristic than the other women in the community) if they had a specific characteristic and the proportion of women in the community who had that characteristic was less than 50%. Women were considered to have less of a characteristic than the community if they did not have a specific characteristic and at least 50% of the women in that community did have that characteristic. Women whose individual response matched the community norm were the reference group (e.g., women who had secondary education, when the proportion of women who had secondary education in the community was greater than or equal 50%).

In addition to the deviance variables, the analysis also considered individual and community level factors. At an individual level, the analysis controlled for age and setting (urban vs. rural). Additional variables included in the analysis were grouped as follows:

#### Socioeconomic characteristics

Previous research suggests that at individual and community levels, income, education, and employment are associated with increased use of modern contraceptives [22, 23, 26, 27]. This may occur through increased knowledge about and access to modern contraceptives as well as an increase in reproductive decision-making power.

Individual-level socioeconomic characteristics included a respondent’s household wealth index score, the woman’s educational attainment, and the woman’s current employment status. Community-level variables measuring socioeconomic characteristics included the proportion of households in the community living in the poorest wealth index, the proportion of women in the community who attained a secondary education or higher, and the proportion of women in the community who were currently employed. Deviance variables measuring socioeconomic characteristics included each respondent’s deviation from each of the community-level socioeconomic characteristics. Women who lived in the poorest wealth index, attained secondary education or higher, or were employed when less than 50% of the women in their communities had these characteristics were all considered to be above their community’s average on these characteristics. Women who did not have these characteristics when at least 50% of the women in the community did have these characteristics were all considered to be below the community’s average on these characteristics.

#### Gender and fertility norms and behaviors

Attitudes towards gender and fertility are important in determining a woman’s motivation and desire to use modern contraceptives and agency in accessing care [28, 29]. Previous research demonstrates the importance of parity, age at marriage, and fertility preferences on modern contraceptive use [11, 27–29]; therefore, these variables represent the gender and fertility norms and behaviors. At an individual-level, variables for gender and fertility norms and behaviors measured age at marriage, parity, and the respondent’s ideal number of children. Community-level gender and fertility norm variables included the community’s mean age at marriage, mean parity, and mean ideal number of children. Deviance variables all considered whether or not an individual woman was at least one standard deviation above or below her community’s mean on these characteristics.

### Analysis

Data were analyzed using STATA 14 software package (College Station, Texas). Twenty-nine separate random-effects logistic regression models were fit (one for each country). All models included all individual-level, community-level, and deviance-level variables, with modern contraceptive use as the dependent variable. A significance level of 0.05 was used to determine statistical significance. The PSU was included as the random intercept, accounting for the hierarchical structure of the data and estimating more accurate standard error. The sigma-mu (random intercept) measured unobserved heterogeneity that exists after controlling for the variables included in the models. To determine the clustered nature of the data, we first fit multi-level models that only included the outcome and the random intercept; a significant random effect signified that the outcome clustered significantly by PSU, illustrating the need to account for the clustering of the data.

When less than 10% of data were missing on all variables, respondents with missing data were not included in their country’s dataset. Variables that were missing data on more than 15% of responses in a country were not included in that country’s analysis. When variables were missing between 10–15%, we determined if data were missing at random; data missing at random were dropped from the dataset, but if data were not missing at random, that variable was instead not included in that country’s analysis.

## Results

Across the 29 countries, the samples ranged from 3,648 respondents in Comoros to 31,079 in Kenya (Table 1). The overall modern contraceptive use (ranging from 5.25% in Guinea to 56.5% in Namibia) and unmet need for modern contraceptives (ranging from 7.9% in Zimbabwe to 27.7% in Liberia) also varied across countries (Table 1).

**Table 1:** Modern contraception use and unmet need across the 29 countries.

	Year	Total Sample Size	% Use of Modern Contraception	% Unmet Need for Modern Contraception
**Lowest quartile unmet need**				
Congo	2011–2012	10,147	17.3	14.3
Kenya	2014	31,079	46.2	12.8
Namibia	2013	9,005	56.5	11.7
Niger	2012	10,202	14.7	14.3
Nigeria	2013	36,018	10.9	12.7
Rwanda	2014–2015	13,353	42.9	12.6
Zimbabwe	2010–2011	9,074	51.8	7.9
**Second quartile of unmet need**				
Cameroon	2011	14,306	15.3	17.5
Ethiopia	2011	11.964	24.2	15.2
The Gambia	2013	9,877	7.9	17.3
Malawi	2010	22,500	39.5	15.1
Senegal	2014	12,379	17.7	16.1
Tanzania	2010	9,889	25.0	16.8
Zambia	2013–2014	11,170	41.4	16.7
**Third quartile of unmet need**				
Burkina Faso	2010	17,087	15.6	20.4
Burundi	2012	8,869	17.4	20.2
Comoros	2012	3,648	13.7	20.6
Gabon	2012	7,899	24.2	20.8
Ghana	2014	9,228	22.4	21.3
Guinea	2012	7,138	5.3	20.1
Mozambique	2011	13,604	14.5	20.8
Sierra Leone	2013	15,846	17.9	20.5
**Highest quartile of unmet need**				
Benin	2011–2012	16,575	8.3	32.6
Côte d’Ivoire	2011–2012	9,191	13.0	22.4
DRC	2013–2014	17,480	7.3	23.5
Liberia	2013	6,610	19.9	27.7
Mali	2012–2013	10,107	12.3	23.3
Togo	2013–2014	9,216	17.0	25.5
Uganda	2011	8,453	24.1	24.5

When less than 10% of data were missing on any of the variables or when 10–15% of responses were missing and determined to be missing at random, respondents with missing data on those variables were not included in their country’s dataset. In Kenya, Ethiopia, Senegal, Comoros, and Guinea, at least one variable needed to be removed from the logistic regression model due to missing data. In all of these cases, more than 15% of responses were missing data or more than 10% were missing and the missing data was found not to be missing at random. Across multiple countries, women who were missing data on the ideal number of children that they wanted were older than women who were not missing data on this variable. The proportion of missing data dropped from the sample ranged from 0% (in Kenya and Senegal) to 8.64% (in Côte d’Ivoire); in 20 of the 29 countries, less than 5% of responses were dropped from the sample due to missing data.

Tables 2 and 3 include the adjusted odds ratios demonstrating the associations between all of the deviance variables for all countries and modern contraceptive use (when adjusted for all other variables in the logistic regression models). In order to understand the context of deviance and modern contraceptive use across countries with different levels of unmet need for modern contraceptives, Tables 2 and 3 are organized by the country’s unmet need for modern contraceptives. Comparisons were not made across countries with different levels of unmet need; however, results are presented in this way in order to assess patterns and understand different country contexts. The Gambia was the only country that did not have any statistically significant deviance variables in the logistic regression model. After controlling for all of the variables in the models, the community-level random effects were significant in all countries except for Guinea.

**Table 2:** Adjusted odds ratios^a^ and 95% confidence intervals examining relationships between socioeconomic characteristic deviance variables and modern contraceptive use from multilevel logistic regression models across 29 countries in sub-Saharan Africa.

	Lives in poorest wealth quintile		Attained secondary education		Employed	
	In poorest wealth quintile, when most of community is not	Not in poorest wealth quintile, when most of community is	Does not have secondary education when most of community does	Has secondary education when most of community does not	Is not employed when most of community is	Is employed when most of community is not
**Lowest quartile unmet need (7.9% to 14.3%)**						
**Congo**	1.00 (0.66, 1.52)	1.02 (0.65, 1.60)	0.83 (0.61, 1.13)	1.19 (0.88, 1.62)	1.27 (0.95, 1.70)	0.78 (0.57, 1.07)
**Kenya**	1.17 (0.90, 1.52)	**1.45 (1.13, 1.87)**	**0.86 (0.74, 1.00)**	**1.21 (1.04, 1.42)**	—	—
**Namibia**	1.11 (0.78, 1.59)	1.11 (0.80, 1.55)	**0.68 (0.52, 0.88)**	**1.41 (1.05, 1.91)**	1.10 (0.89, 1.35)	**0.79 (0.65, 0.96)**
**Niger**	0.74 (0.32, 1.70)	1.37 (0.64, 2.93)	0.52 (0.27, 1.01)	1.13 (0.59, 2.13)	0.68 (0.42, 1.09)	1.14 (0.75, 1.76)
**Nigeria**	0.50 (0.19, 1.28)	0.83 (0.42, 1.64)	0.86 (0.68, 1.09)	**1.40 (1.11, 1.77)**	1.03 (0.82, 1.28)	0.94 (0.75, 1.18)
**Rwanda**	1.00 (0.67, 1.50)	**1.46 (1.01, 2.13)**	0.94 (0.68 1.29)	1.03 (0.74, 1.44)	**1.57 (1.09, 2.27)**	**0.60 (0.41, 0.85)**
**Zimbabwe**	1.07 (0.72, 1.55)	1.16 (0.83 1.63)	0.93 (0.71 1.21)	1.22 (0.91, 1.64)	0.99 (0.76, 1.29)	0.96 (0.76, 1.22)
**Second quartile of unmet need (15.1% to 17.5%)**						
**Cameroon**	1.20 (0.53, 2.76)	0.83 (0.39, 1.71)	1.10 (0.85, 1.42)	1.20 (0.93, 1.54)	1.11 (0.90, 1.38)	0.90 (0.71, 1.13)
**Ethiopia**	0.69 (0.40, 1.20)	**2.10 (1.20, 3.67)**	0.97 (0.59, 1.61)	1.57 (0.95, 2.61)	0.98 (0.71, 1.37)	1.31 (0.96, 1.80)
**The Gambia**	0.87 (0.46, 1.66)	1.13 (0.60, 2.14)	1.01 (0.62, 1.63)	0.96 (0.59, 1.58)	1.31 (0.81, 2.13)	0.91 (0.60, 1.38)
**Malawi**	1.12 (0.86, 1.47)	0.90 (0.70, 1.16)	0.81 (0.63, 1.04)	1.11 (0.87, 1.41)	0.89 (0.77, 1.04)	1.01 (0.87, 1.17)
**Senegal**	1.35 (0.82, 2.22)	1.04 (0.63, 1.72)	**0.56 (0.37, 0.84)**	**1.97 (1.23, 3.15)**	0.85 (0.62, 1.16)	1.13 (0.84, 1.50)
**Tanzania**	1.34 (0.85, 2.13)	**0.52 (0.34, 0.82)**	1.04 (0.66, 1.65)	**1.75 (1.10, 2.78)**	1.56 (0.99, 2.45)	0.90 (0.58, 1.40)
**Zambia**	1.30 (0.94, 1.74)	1.02 (0.75, 1.38)	0.87 (0.70, 1.08)	1.06 (0.84, 1.33)	**0.76 (0.62, 0.93)**	0.98 (0.81, 1.19)
**Third quartile of unmet need (20.1% to 21.3%)**						
**Burkina Faso**	0.83 (0.52, 1.31)	**1.62 (1.01, 2.61)**	0.93 (0.62, 1.40)	1.15 (0.79, 1.68)	0.88 (0.62, 1.24)	1.03 (0.72, 1.49)
**Burundi**	0.91 (0.47, 1.76)	0.64 (0.34, 1.21)	0.97 (0.53, 1.75)	0.63 (0.37, 1.08)	0.99 (0.63, 1.54)	1.06 (0.69, 1.64)
**Comoros**	0.55 (0.22, 1.40)	1.32 (0.64, 2.74)	1.15 (0.73, 1.81)	0.71 (0.41, 1.25)	1.39 (0.82, 2.35)	**0.48 (0.31, 0.76)**
**Gabon**	1.11 (0.70, 1.75)	1.13 (0.71, 1.81)	0.83 (0.57, 1.21)	1.05 (0.73, 1.53)	1.11 (0.82, 1.51)	0.93 (0.69, 1.27)
**Ghana**	0.80 (0.45, 1.44)	1.09 (0.61, 1.94)	0.79 (0.56, 1.11)	**1.61 (1.13, 2.27)**	1.34 (0.81, 2.21)	0.99 (0.62, 1.58)
**Guinea**	1.62 (0.52, 5.04)	0.45 (0.16, 1.26)	0.74 (0.32, 1.72)	1.02 (0.42, 2.46)	1.13 (0.50, 2.53)	0.92 (0.46, 1.83)
**Mozambique**	0.98 (0.49, 1.94)	1.07 (0.54, 2.12)	1.11 (0.82, 1.49)	0.96 (0.73, 1.27)	**1.34 (1.02, 1.75)**	0.79 (0.61, 1.02)
**Sierra Leone**	1.15 (0.77, 1.71)	1.43 (0.97, 2.10)	0.98 (0.73, 1.32)	**1.52 (1.15, 2.01)**	1.22 (0.96, 1.54)	0.81 (0.63, 1.03)
**Highest quartile of unmet need (22.4% to 32.6%)**						
**Benin**	0.96 (0.54, 1.71)	1.31 (0.74, 2.30)	0.95 (0.61, 1.49)	0.93 (0.62, 1.39)	0.90 (0.62, 1.29)	0.96 (0.66, 1.42)
**Côte d’Ivoire**	0.88 (0.52, 1.48)	0.97 (0.56, 1.69)	0.98 (0.66, 1.45)	1.29 (0.90, 1.85)	0.97 (0.68, 1.38)	0.79 (0.54, 1.16)
**DRC**	**0.53 (0.29, 0.95)**	1.55 (0.89, 2.70)	1.27 (0.81, 1.98)	1.07 (0.71, 1.62)	1.22 (0.86, 1.74)	1.03 (0.72, 1.47)
**Liberia**	0.98 (0.61, 1.58)	0.93 (0.58, 1.51)	0.88 (0.53, 1.44)	1.49 (0.92, 2.40)	0.92 (0.63, 1.34)	1.33 (0.93, 1.91)
**Mali**	**2.11 (1.09, 4.07)**	0.52 (0.26, 1.06)	0.89 (0.55, 1.14)	1.16 (0.74, 1.81)	0.85 (0.59, 1.23)	1.15 (0.82, 1.62)
**Togo**	0.67 (0.40, 1.12)	1.48 (0.90, 2.42)	0.98 (0.69, 1.40)	1.13 (0.81, 1.57)	0.89 (0.58, 1.35)	0.97 (0.63, 1.50)
**Uganda**	1.11 (0.68, 1.81)	0.71 (0.44, 1.15)	0.86 (0.62, 1.19)	1.60 (1.17, 2.20)	1.17 (0.85, 1.61)	0.83 (0.60, 1.15)

^a^All odds ratios are adjusted for age, living in a rural setting, and all individual-level variables, community-level variables, and deviance-level variables addressing socioeconomic characteristics (poverty, education, employment) and gender and fertility norms and behaviors (parity, age at marriage, fertility preferences).

**Table 3:** Adjusted odds ratios^a^ and 95% confidence intervals examining relationships between gender and fertility norms and behavior deviance variables and modern contraceptive use from multilevel logistic regression models across 29 countries in sub-Saharan Africa.

	Parity		Age at marriage		Ideal number of children	
	Lower parity than community	Higher parity than community	Younger age at marriage than community	Older age at marriage than community	Wants fewer children than community	Wants more children than community
**Lowest quartile unmet need (7.9% to 14.3%)**						
**Congo**	1.24 (1.00, 1.56)	0.88 (0.66, 1.18)	1.08 (0.86, 1.35)	**1.30 (1.10, 1.53)**	**0.79 (0.63, 0.98)**	1.13 (0.85, 1.49)
**Kenya**	1.10 (0.94, 1.29)	0.96 (0.83, 1.11)	**0.86 (0.77, 0.96)**	**0.65 (0.60, 0.70)**	—	—
**Namibia**	1.04 (0.86, 1.26)	1.16 (0.91, 1.48)	1.14 (0.91, 1.44)	1.10 (0.98, 1.23)	0.89 (0.74, 1.06)	0.86 (0.70, 1.06)
**Niger**	1.01 (0.72, 1.43)	1.10 (0.85, 1.42)	**0.72 (0.57, 0.93)**	1.30 (0.98, 1.73)	1.14 (0.88, 1.49)	0.85 (0.63, 1.15)
**Nigeria**	0.95 (0.79, 1.15)	1.18 (0.98, 1.43)	0.99 (0.85, 1.15)	**1.52 (1.36, 1.70)**	0.96 (0.79, 1.18)	0.94 (0.77, 1.15)
**Rwanda**	1.48 (0.85, 2.58)	1.13 (0.92, 1.38)	1.00 (0.81, 1.22)	**0.66 (0.58, 0.74)**	1.05 (0.86, 1.27)	0.97 (0.79, 1.19)
**Zimbabwe**	1.07 (0.79, 1.44)	0.95 (0.75, 1.19)	0.89 (0.73, 1.10)	**0.80 (0.69, 0.93)**	**0.81 (0.66, 0.99)**	0.92 (0.74, 1.13)
**Second quartile of unmet need (15.1% to 17.5%)**						
**Cameroon**	**0.78 (0.63, 0.98)**	1.18 (0.92, 1.53)	0.96 (0.79, 1.18)	**1.18 (1.02, 1.35)**	1.06 (0.86, 1.31)	0.89 (0.69, 1.15)
**Ethiopia**	0.95 (0.70, 1.30)	1.03 (0.81, 1.30)	0.89 (0.75, 1.06)	0.96 (0.79, 1.16)	—	—
**The Gambia**	0.77 (0.29, 2.04)	1.04 (0.70, 1.54)	0.81 (0.58, 1.14)	1.00 (0.76, 1.32)	1.00 (0.69, 1.46)	0.91 (0.59, 1.41)
**Malawi**	0.89 (0.73, 1.10)	1.02 (0.88, 1.19)	**0.85 (0.76, 0.95)**	**0.82 (0.73, 0.93)**	0.91 (0.80, 1.04)	1.01 (0.88, 1.16)
**Senegal**	1.15 (0.65, 2.03)	1.04 (0.82, 1.31)	**0.81 (0.66, 0.98)**	1.02 (0.85, 1.22)	—	—
**Tanzania**	1.34 (0.99, 1.80)	1.03 (0.79, 1.34)	1.22 (0.98, 1.52)	1.04 (0.88, 1.25)	1.21 (0.95, 1.55)	0.80 (0.61, 1.06)
**Zambia**	0.86 (0.65, 1.14)	1.10 (0.93, 1.31)	**0.85 (0.73, 0.98)**	1.00 (0.87, 1.17)	1.04 (0.87, 1.26)	0.85 (0.71, 1.00)
**Third quartile of unmet need (20.1% to 21.3%)**						
**Burkina Faso**	0.90 (0.71, 1.13)	1.09 (0.89, 1.34)	1.12 (0.93, 1.35)	0.98 (0.82, 1.16)	1.08 (0.84, 1.38)	1.04 (0.81, 1.35)
**Burundi**	**0.37 (0.15, 0.95)**	1.10 (0.78, 1.56)	0.93 (0.69, 1.25)	**0.79 (0.63, 0.99)**	0.80 (0.59, 1.08)	0.93 (0.65, 1.33)
**Comoros**	0.51 (0.21, 1.27)	0.81 (0.52, 1.26)	0.99 (0.70, 1.40)	0.80 (0.55, 1.14)	—	—
**Gabon**	1.14 (0.90, 1.45)	**1.51 (1.06, 2.16)**	1.15 (0.88, 1.52)	1.06 (0.90, 1.26)	0.88 (0.69, 1.11)	0.75 (0.54, 1.04)
**Ghana**	0.99 (0.75, 1.31)	0.81 (0.62, 1.06)	0.81 (0.64 (1.02)	0.89 (0.75, 1.06)	0.93 (0.73, 1.19)	0.80 (0.60, 1.06)
**Guinea**	**2.00 (1.02, 3.91)**	1.16 (0.71, 1.88)	0.90 (0.62, 1.32)	00.83 (0.55, 1.28)	—	—
**Mozambique**	0.83 (0.64, 1.09)	1.16 (0.89, 1.52)	0.99 (0.79, 1.24)	1.01 (0.86, 1.19)	**0.66 (0.52, 0.84)**	0.97 (0.75, 1.25)
**Sierra Leone**	0.98 (0.82, 1.16)	0.97 (0.79, 1.19)	1.20 (1.00, 1.44)	**1.73 (1.50, 1.99)**	0.94 (0.78, 1.12)	0.77 (0.62, 0.95)
**Highest quartile of unmet need (22.4% to 32.6%)**						
**Benin**	1.20 (0.95, 1.51)	1.04 (0.79, 1.37)	0.82 (0.64, 1.05)	1.19 (0.99, 1.43)	**0.75 (0.58, 0.96**)	0.98 (0.75, 1.29)
**Côte d’Ivoire**	0.92 (0.69, 1.23)	1.06 (0.78, 1.45)	0.89 (0.67, 1.18)	**1.25 (1.04, 1.51)**	0.88 (0.68, 1.14)	**0.68 (0.48, 0.95)**
**DRC**	1.11 (0.84, 1.48)	1.16 (0.84, 1.62)	0.99 (0.78, 1.27)	**1.25 (1.02, 1.52)**	1.02 (0.78, 1.34)	0.77 (0.57, 1.05)
**Liberia**	**0.64 (0.42, 0.99)**	1.14 (0.87, 1.48)	0.83 (0.65, 1.06)	0.89 (0.70, 1.13)	0.79 (0.45, 1.39)	**3.69 (1.20, 11.40)**
**Mali**	0.96 (0.68, 1.35)	1.09 (0.80, 1.49)	0.96 (0.74, 1.24)	0.93 (0.74, 1.19)	1.02 (0.77, 1.36)	0.91 (0.65, 1.27)
**Togo**	**0.58 (0.44, 0.77)**	1.14 (0.87, 1.49)	1.20 (0.92, 1.55)	**1.24 (1.03, 1.49)**	0.94 (0.74, 1.21)	0.85 (0.64, 1.14)
**Uganda**	1.12 (0.83, 1.52)	0.90 (0.71 1.13)	1.10 (0.89, 1.35)	**0.80 (0.65, 0.96)**	1.13 (0.88, 1.45)	1.12 (0.85, 1.49)

^a^ All odds ratios are adjusted for age, living in a rural setting, and all individual-level variables, community-level variables, and deviance-level variables addressing socioeconomic characteristics (poverty, education, employment) and gender and fertility norms and behaviors (parity, age at marriage, fertility preferences).

### Socioeconomic characteristics

Table 2 presents results for deviance variables representing socioeconomic characteristics. When examining the relationships between deviance variables and contraceptive use, some clear patterns emerged across different countries. In three countries (Kenya, Namibia, and Senegal) women who did not have secondary education when more than 50% of the women in the community did were less likely to report using modern contraceptives. At the same time, in these same countries and in four additional countries (Nigeria, Tanzania, Ghana, and Sierra Leone), women who attained at least a secondary education when less than 50% of the women in their community did were more likely to report using modern contraceptives.

Results for the other socioeconomic characteristics varied more across countries. Women who were employed when most of the community was not were less likely to report using modern contraceptives; this relationship was consistent across three countries (Namibia, Rwanda, and Comoros). However, when examining how not being employed when most of the community was associated with modern contraceptive use, there was some variation across countries. In Zambia, being below the community average on employment was associated with a decrease in modern contraceptive use, but in Rwanda and Mozambique, this was associated with an increase in modern contraceptive use. There was even more variation when examining wealth index; seven countries having significant findings on wealth index, but the direction of these relationships was inconsistent across countries.

### Gender and fertility norms and behaviors

Table 3 presents results for deviance variables representing gender and fertility norms and behaviors. Deviance on age at marriage was statistically associated with modern contraceptive use in more countries than any other variable, with more than half (n = 16) of the countries having significant results on these variables. The direction of these relationships was not always consistent across countries, though. Marrying younger than other women in the community was consistently associated with a decrease in modern contraceptive use in five countries (Kenya, Niger, Malawi, Senegal, and Zambia). However, marrying older than other women in the community was associated with an increase in modern contraceptives some countries (n = 7), but was associated with a decrease in modern contraceptive in other countries (n = 6).

Deviating from the ideal number of children that a woman wanted was statistically significant in six countries (Congo, Zimbabwe, Guinea, Benin, Côte d’Ivoire, and Liberia). Across all of these countries except one (Liberia), being deviant on this variable in either direction (i.e., wanting fewer or more children than other women in the community) was associated with a decrease in modern contraceptive use. However, in Liberia, wanting more children than other women in the community was associated with having 3.69 times the odds of reporting modern contraceptive use (p = 0.023).

Most countries that had a significant relationship between deviance on parity found that having fewer children than other women in the community was associated with an increase in modern contraceptive use, while having more children was associated with an increase. The one exception to this was also in Guinea, where having fewer children than other women in the community was associated with twice the odds of reporting modern contraceptive use (p = 0.043).

## Discussion

Findings suggest that deviance when women (and their families) deviate from their communities on issues such as a woman’s education or the age that a woman gets married, this may influence autonomy and decision-making around modern contraceptive use. Women who had more education than other women in their community were consistently more likely to report modern contraceptive use, while women who had less education than other women in their community were consistently less likely to report modern contraceptive use. As a socioeconomic characteristic, the experience of being deviant and having more education than other women in the community may be related to socioeconomic advantage. Within an environment where most women do not attain a secondary education, attaining this level of education may represent additional socioeconomic privileges that could help to increase a woman’s ability to access modern contraceptives. This experience of socioeconomic advantage and having more education may also be directly linked with experiences of autonomy as well as fertility preferences. When women have more education, they may have more decision-making power within their families [28, 30, 31]. In addition, women who attain a secondary education may come from a family that values women’s autonomy and empowerment [32, 33]. Furthermore, women who have more education than other women in the community may start childbearing at a later age or may have additional goals for education that may conflict with childbearing; in an environment where most women do not have secondary education, there may be fewer opportunities to have children while simultaneously attaining education.

Another deviance variable that was statistically significant across many countries was age at marriage. Marrying at a younger age than other women in the community was consistently associated with reduced use of modern contraceptives. In this case, women who marry younger than other women in their community may have less autonomy to make decisions around modern contraceptive use [34, 35]. It is also possible that women who marry younger are expected to have more children and therefore are not using modern contraceptives [34, 35]. Results were inconsistent among women who married older than other women in their communities. In some countries, this was associated with an increase in modern contraceptive use and in others it was associated with a decrease. If women are trying to match the fertility norms of their communities [9], it is possible that marrying at an age that is later than the community norm may result in the desire to have children right away, and therefore may reduce the likelihood of using modern contraceptive use. At the same time, marrying older than other women in the community may also be indicative of women having values outside of the family (e.g., values related to education or employment) or having increased decision-making power, which may result in women choosing to use modern contraceptives [34, 35].

### The value of a deviance framework

Findings demonstrate how a deviance framework can build on the socio-ecological model, demonstrating how interacting with one’s environment may influence modern contraceptive use [17]. The socioeconomic characteristics and gender and fertility norms and behaviors used in this deviance analysis have all been previously identified as being associated with modern contraceptive use at an individual and/or community level [10, 11, 22–24, 26–29]. However, even after controlling for all of the variables at individual and community levels, the deviance variables were still associated with modern contraceptive use. It is not only an individual woman’s expeirence or the community in which she resides that matters, but rather the way in which she interacts with her environment.

The associations between deviance on fertility preferences and practices and modern contraceptive use provide good examples of how the relationship between an individual woman and her community matters. Across countries, women who wanted fewer children than other women in their communities were less likely to report using modern contraceptives. In addition, with the exception of results from one country, having a lower parity than other women the community was consistently associated with less use of modern contraceptives. At an individual level, wanting or having fewer children may be expected to be associated with an increase in modern contraceptive use [36]; women with lower fertility preferences and/or parity may choose to have fewer children and therefore may be more likely to use modern contraceptives. However, previous research suggests that women may attempt to match their own fertility ideals to the norms of the places where they live [9]. Therefore, women who want and/or have fewer children than other women in their communities may be less likely to use modern contraceptives in order to better fit into community norms.

### Country context matters

For most of the deviance variables, results varied across countries. In some cases, the same deviance variable was associated with an increase in modern contraceptive use in one country, but a decrease in another, while not having a significant association in other countries. This suggests that the country context matters when considering the role that deviance places on modern contraceptive use. The overall country context may shape experiences of deviating from community norms. Further country-specific research is warranted to better understand how deviance from community norms facilitates experiences of health and modern contraceptive use in specific environments.

Though statistical comparisons were not made, the level of unmet need in a country did not seem to matter with the relationship between the deviance variables and modern contraceptive use. The overall level of unmet need and the overall among of modern contraceptive use is expected to shape an individual’s likelihood of using modern contraceptives. Women living in countries with less unmet need and a greater use of modern contraceptives are expected to be more likely to report using modern contraceptives themselves; the opposite is true for women living in countries with a greater unmet need and less overall use of modern contraceptives. Therefore, it is surprising that no clear patterns emerged across countries with different levels of unmet need for modern contraceptives. More research is warranted to explore these relationships.

### Implications

Even though findings varied across countries, these results still have programmatic as well as research implications. Additional qualitative research may be especially useful to better understand experiences of deviance within specific country contexts. Once these experiences are better understood, experiences of positive and negative deviance can help to inform public health interventions.

When women who are positive deviant on socioeconomic and gender and fertility norm behaviors also demonstrate increased use of modern contraceptive use and/or a lower unmet need for modern contraceptives, this demonstrates the importance of focusing on women’s autonomy and socioeconomic factors as a way to help improve modern contraceptive use. Further understanding why and how women are deviant and what about being deviant helps to improve modern contraceptive use can be useful for determining strategies for interventions. At the same time, understanding the experiences of women who are negatively deviant on socioeconomic characteristics and gender and fertility norms and behaviors can be useful for informing public health practitioners how to reach these potentially vulnerable populations. Often, public health interventions are distributed at a community-level; however, more attention may need to be given to specific and potentially harder-to-reach women within communities in order to help them to improve health outcomes and avoid adverse health outcomes.

### Limitations and strengths

There were some limitations in this study. These findings were cross-sectional, so no causal pathways could be concluded. It is possible that aggregate (i.e., community-level) variables do not fully capture community effects; however, the DHS does not collect community-level data across all 29 countries included in this analysis, so community-level deviance variables were limited to the use of aggregate variables. Furthermore, PSUs were used to define communities. Each PSU consists of 20–30 households in a geographical region, limiting the definition of community. PSUs are artificial sampling units and may not necessarily reflect what a person thinks of as their community. In addition, regions varied in size and some PSUs contained fewer than ten respondents. However, analysis showed that the outcomes did vary significantly by PSU, suggesting these areas have an influence on outcomes, and model checking found no instability due to small number of respondents in some PSUs. Similar methods have been used in other analyses using DHS data to examine community effects [11, 37]. In addition, across all countries in the study, if data were missing on less than 10% of responses, missing data were dropped from the dataset. This may create selection bias; however, in order to reduce this, variables missing more than 10% of data with data not missing at random, or variables missing more than 15% of data were removed from the analysis in that country. While this reduced selection bias, it also resulted in five countries having slightly different regression models (Kenya, Ethiopia, Senegal, Comoros, and Guinea) than all other countries. Furthermore, despite controlling for individual-level, community-level, and deviance variables, there was still unobserved heterogeneity in most of the models. Other important factors not collected by DHS may explain the community-level random effects. Most notably, in most countries, DHS does not collect data on the existence or quality of health services, which may play a very important role in a woman’s ability to access and use modern contraceptives.

## Conclusions

Taken together, these findings demonstrate that using a deviance approach can provide additional knowledge when trying to understand who is using modern contraceptives (and who is not). This approach is especially useful when considering factors related to women’s autonomy (such as a woman’s education or age at marriage). A deviance lens is important as it examines the interplay between an individual woman and her community. This study highlights the relationships between modern contraceptive use, sociodemographic characteristics, and gender and fertility norms and behaviors, when an individual’s experiences of these factors are inconsistent with the experiences of other women in their community. The current study recognizes the importance of moving beyond examining only individual- and community-level determinants of modern contraceptive use, and highlights that it may also be important to understand not only the context in which individuals live, but also the relative position of individuals in their community. Since results differed across countries, this study also highlights how a country’s specific context can play a role in the relationship between deviance and modern contraceptive use across countries. When understood within country-specific contexts, these experiences of deviance may be able to provide insight into additional programmatic needs and inform interventions that aim to increase the use of modern contraceptives.
